# Feasibility and acceptability of injectable artesunate for the treatment of severe malaria in the Democratic Republic of Congo

**DOI:** 10.1186/s12936-015-1072-x

**Published:** 2016-01-08

**Authors:** Henry M. T. Ntuku, Gianfrancesco Ferrari, Christian Burri, Antoinette K. Tshefu, Didier M. Kalemwa, Christian Lengeler

**Affiliations:** Kinshasa School of Public Health, Kinshasa, Democratic Republic of the Congo; Swiss Tropical and Public Health Institute, Basel, Switzerland; University of Basel, Basel, Switzerland

**Keywords:** Malaria, Severe malaria, Injectable artesunate, Injectable quinine, Feasibility, Democratic Republic of the Congo

## Abstract

**Background:**

The Democratic Republic of the Congo (DRC) changed its national policy for the treatment of severe malaria in both children and adults in 2012 from intravenous quinine to injectable artesunate. The country is now planning to deploy nationwide injectable artesunate as the preferred treatment for the management of severe malaria. To support this process, the feasibility and acceptability of the use of injectable artesunate in the context of the DRC was assessed, from the perspective of both health care providers and patients/caretakers.

**Methods:**

Questionnaires and observations were used to collect information from health care providers and patients/caretakers in eight health facilities in the Province of Kinshasa and in the Province of Bas-Congo.

**Results:**

A total of 31 health care providers and 134 patients/care takers were interviewed. Seventy five percent (75 %) of health care providers found it less difficult to prepare injectable artesunate compared to quinine. None of them encountered problems during preparation and administration of injectable artesunate. The large majority of care providers (93 %) and patients/caretakers (93 %) answered that injectable artesunate took less time than quinine to cure the symptoms of the patients. 26 (84 %) health care providers reported that the personnel workload had diminished with the use of injectable artesunate. 7 (22.6 %) health workers reported adverse drug reactions, of which a decrease in the haemoglobin rate was the most common (71.4 %). All care providers and the vast majority of patients/caretakers (96 %, N = 128) were either satisfied or very satisfied with injectable artesunate.

**Conclusions:**

These findings show that the use of injectable artesunate for the treatment of severe malaria is feasible and acceptable in the context of DRC, with appropriate training of care providers. Both care providers and patients/caretakers perceived injectable artesunate to be effective and safe, thus promoting acceptability.

## Background

In the Democratic Republic of the Congo (DRC), malaria is one of the leading causes of death in children under 5 years of age, with an estimated 9,000,000 cases and 22,000 deaths reported in 2012 [1]. As a result, the DRC is the second country in the world in terms of burden of malaria [2, 3]. For severe malaria, the case fatality is reaching 10 % [4]. Severe malaria is obviously a medical emergency, and reducing its burden is currently the highest priority of malaria control, as evidenced by the Roll Back malaria (RBM) target of near-zero deaths by 2015 [5].

For the management of severe malaria cases, comparative clinical trials between quinine and injectable artesunate have demonstrated that the treatment with artesunate was associated with a substantial reduction of case fatality in both children and adults [6–8]. In addition, intravenous artesunate offers a number of programmatic advantages over quinine in terms of not requiring rate-controlled infusion or cardiac monitoring [9]. These results led to a change in the WHO guidelines for the treatment of severe malaria in 2011, recommending intravenous artesunate as the preferred treatment for severe malaria in children and adults [10]. As a result of this change, an additional 195,000 deaths could be averted every year in Africa [11]. Following the new WHO guidelines, the National Malaria Control Programme (NMCP) of the DRC changed the national policy for the treatment of severe malaria in both children and adults from intravenous quinine to injectable artesunate in 2012 [12]. However, this policy change requires a number of clinical and operational adaptations, as quinine has been the treatment of choice for many decades. The national strategic plan set up an implementation period of 3 years to scale up injectable artesunate.

The handling of injectable artesunate is reported to be easier compared to quinine, however a number of operational issues such as dosing and preparation of the drug may hinder its use.

One important element for a successful transition, besides logistical aspects, is ensuring that there is a high acceptability of the new treatment by the health care providers, as well as by the patients. Finally, there is also a need to determine the perceived effectiveness and safety of the new treatment. These factors are a prerequisite for achieving a successful rollout and therefore high public health impact. This study investigates the feasibility and acceptability of the use of injectable artesunate in the context of the DRC, to identify arising issues and propose solutions before the start of the national rollout.

Although a number of studies have investigated the efficacy of injectable artesunate for the treatment of severe malaria as well as some issues related to its use [13–15], none has focused so far on the feasibility of the implementation of the new IV/IM anti-malarial drug from the perspective of care providers, as well as its acceptability from the perspective of patients/caretakers.

## Methods

### Study sites

This study was conducted as part of the MATIAS study (Treatment of severe malaria—an operational comparative study for the treatment of severe malaria between quinine and artesunate in Hospitals and Health Centres of Kinshasa and Bas Congo province). The MATIAS study was a non-controlled operational comparative study conducted in children and adults admitted with severe malaria to hospital and health centres [16].

**Fig. 1:**
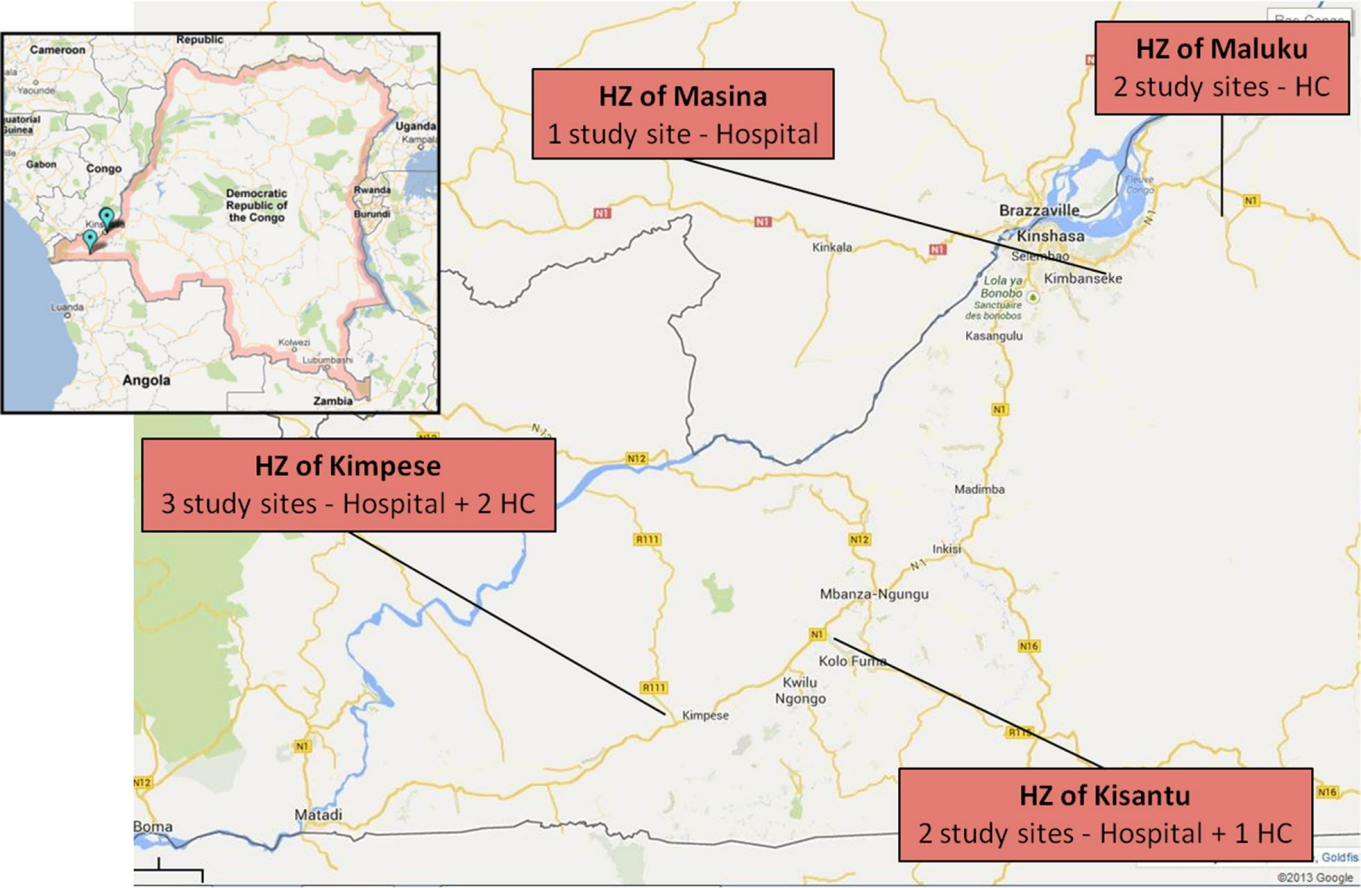
Map showing the location of the study sites. *HZ* Health Zone; *HC* Health Centre

The study was implemented in eight health facilities (three hospitals and five health centres) in Greater Kinshasa, the capital of the DRC (Referral Hospital Roi Baudoin, Health Centre Bita, Health Centre Menkao) and in the Province of Bas-Congo (Referral Hospital Saint Luc Kisantu, Health Centre Ngeba, Referral hospital of Kimpese, Health Centre Ceco, Health Centre La Famille). Figure 1 shows the location of the study sites. Selected health facilities were representative of typical health facilities in the country including a large public health hospital; a medium-sized, non-profit, missionary hospital; a medium-sized, government hospital (Centre Hospitalier Roi Baudouin) and rural health centres.

Kinshasa sites serve urban and semi-rural populations, whereas the Bas-Congo sites serve a largely rural population. All sites are hyper to holoendemic for malaria and transmission is perennial with seasonal variation [17, 18]. At the time the study started, injectable artesunate had not been deployed to public health facilities and was not available in the private sector.

The MATIAS study was conducted in two consecutive phases. In the first phase, in the eight selected study sites, a target number of 350 patients were recruited over 3 months, from October 2012 to January 2013, with intravenous quinine as the treatment drug. In the second phase, following the introduction of injectable artesunate, the same target number of patients was recruited over the 3 months period, from April to July 2013. A three-month interval was kept between the two phases in order to train the healthcare providers from the study sites in the preparation and administration of the new drug.

With regard to the use of injectable artesunate in hospitals, clinicians were responsible for prescribing the drug, specifying the dose needed and the schedule of dosing and evaluating patients’ progress while nurses prepared and administered the drug. In health centres, nurses were responsible for all aspects of drug use.

The MATIAS study included four key components: (1) clinical assessment of patients, (2) a time and motion study, (3) financial costs, (4) feasibility and acceptability assessments through providers and patients/caretakers questionnaires. The results of the first three components are reported elsewhere [16], while the results of the fourth component are reported here.

All interviews for the feasibility and acceptability assessment were conducted during the second phase (artesunate phase) between April and July 2013, since the aim was chiefly to compare assessments of quinine versus artesunate.

Participants belonged to two groups with separate questionnaires: (1) Health care providers who prescribed or administered injectable artesunate during the MATIAS study and whose verbal consent was obtained. A purposive sample of four health care providers per health facility was interviewed, which represents the mean number of personnel trained in the use of injectable artesunate per health facility. (2) patients/caretakers of patients who were treated with injectable artesunate in each study site. A convenience sample of one-third of all patients/caretakers of patients attending follow up visits was interviewed. Patients/caretakers of patients were eligible for interview if they had personal past experience with quinine treatment or have taken care of another member of the family in the past treated with quinine and they must give verbal consent. Patients or caretakers of patients were randomly selected.

### Training and implementation of injectable artesunate

**Fig. 2:**
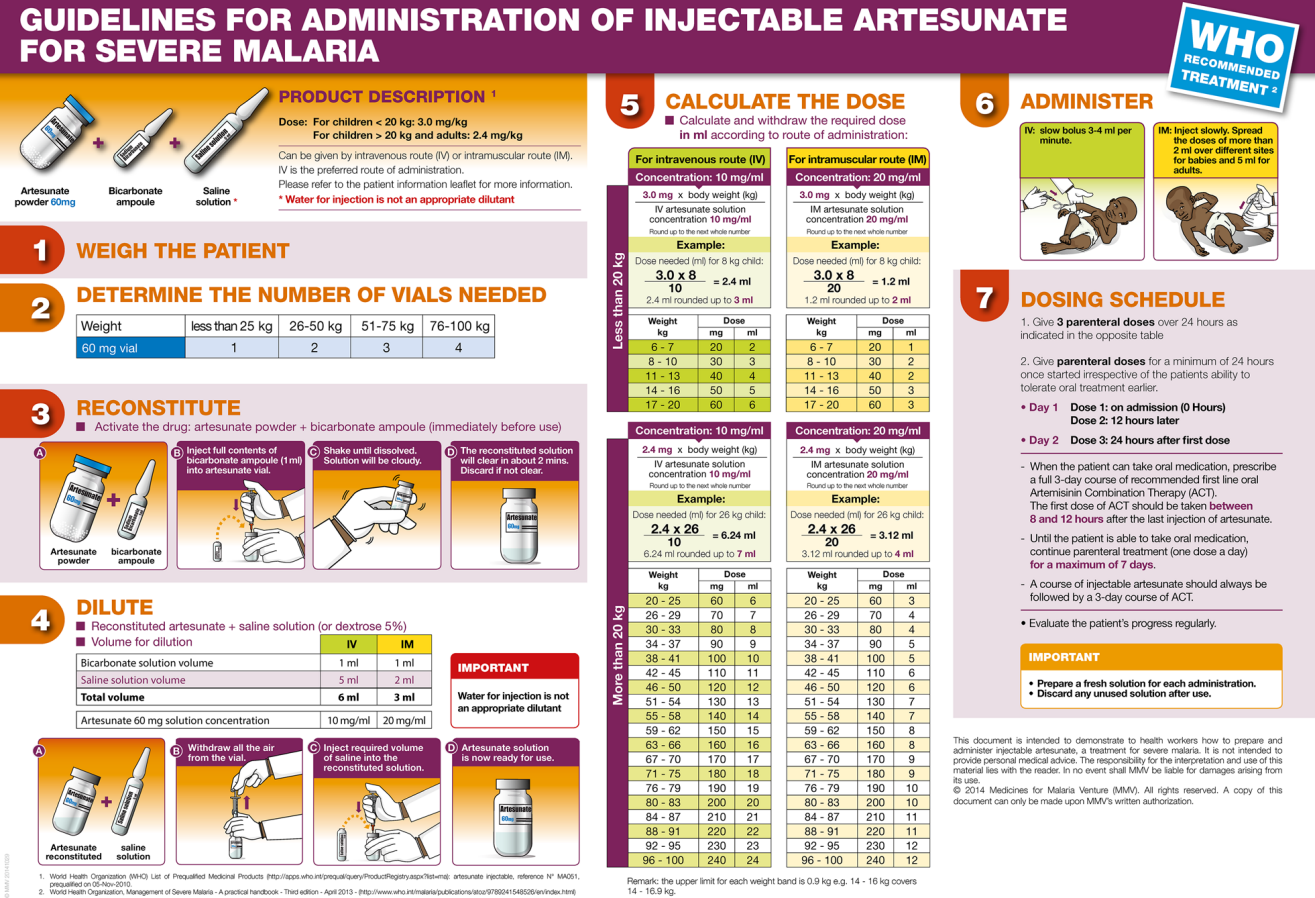
Injectable artesunate job aid (poster)

In preparation for the first part of the study (quinine treatment), a three-day training on study procedures was given to all investigators and staff involved in the patient’s clinical management in each hospital and health centre. The training included an update of knowledge on malaria diagnosis and management. Before starting the second phase, a two-day training on the preparation and administration of injectable artesunate was given to all staff involved in clinical management in the study sites. These sessions used a new training tool kit developed and provided by the Medicines for Malaria Venture (MMV) product development partnership. This kit consisted of a very detailed user guide; an explicit and straightforward job aid (Fig. 2), and a practical training video. Prior to patient recruitment, health care providers were allowed some time to become familiar with the handling of the new drug under supervision.

Injectable artesunate (Guilin Pharmaceutical Co, Ltd, Shanghai, China) was packed in boxes each containing one vial of 60 mg of artesunate powder for injection, one ampoule of sodium bicarbonate and one ampoule of sodium chloride. The following steps were required prior to drug injection: (1) calculation of the number of vials required based on patient weight, (2) reconstitution of artesunate solution with sodium bicarbonate solution, (3) dilution of the solution with sodium chloride.

Artesunate was given intravenously at a dose of 2.4 mg/kg bodyweight at 0, 12 and 24 h, and then once a day until the patient was able to take oral treatment. In line with the WHO recommendations [9], parenteral treatment was given for a minimum of 24 h, irrespective of the patient’s ability to tolerate oral medication. After completion of the injectable treatment, the patient was given a full course of the recommended oral artemisinin-based combination therapy, AS-AQ or AL. Alternatively, parenteral artesunate was given for a maximum of 7 days, until oral treatment could be taken reliably.

Patients were followed up at day 7, day 14, day 21 and day 28 after discharge. Artesunate was provided free of charge by the manufacturer (Guilin Pharmaceuticals, Shanghai, PDR China) while the costs of quinine were covered by the study. In each study site, patients were managed by local clinicians (hospitals) or nurses (health centres), while the research team carried out a weekly supervision at each study site throughout the duration of the study. The NMCP provided policy support. All authorizations for drug importation were obtained from the Ministry of Health through the National Drug Authority. All relevant authorities were actively involved in the planning of the study and preliminary results of the study were shared and discussed during stakeholders meetings. Unpublished preliminary results of the study were used by the NMCP to develop training manuals for healthcare providers and communication tools in prevision of the deployment of injectable artesunate.

### Data collection

Two questionnaires were used to collect data. Interviews were conducted by nine trained interviewers recruited from the local community. Two of them were physicians, four were nurses and three were social workers. The two physicians were recruited from Kinshasa and conducted interviews with all study physicians. Two nurses and one social worker were recruited in Kinshasa and conducted interviews respectively with nurses and patients/caretakers in Kinshasa sites. Two nurses and two social workers were recruited in Bas Congo and conducted interviews respectively with nurses and patients/caretakers in Bas Congo sites. These interviewers were supervised by study field scientists. A three-day training was given to all interviewers prior to data collection. The training included familiarization with the study tools and practicing interviews. Basic techniques of probing and recording responses were also discussed during the training. Interview guides were developed and pre-tested prior to use.

Interviews with *care providers* focused on ease of application and drug handling, perceived safety of the treatment, quality of the patient management, perception of old versus new treatment on staff work load, and level of satisfaction with the new treatment. The core questions of the interviews compared injectable artesunate and quinine. While obviously there could have been a recall bias due to the fact that the interviews were done during the artesunate phase of the study, about 3–6 months after the quinine phase, this should not have been too much of an issue since quinine has been used for decades in the DRC, and all health care providers were very familiar with its use.

Interviews with *patients/caretakers* took place during the follow up visits and focused on the perception of the effectiveness and safety of injectable artesunate, especially with regard to adverse events. Here, recall bias could have been more of an issue since patients were less familiar with quinine adverse events. In order to minimize this problem, one inclusion criterion for the interviews of patients/caretakers was a past experience with quinine treatment, either for themselves or for one member of the family.

According to the interviewee’s preference, interviews were conducted in French, the official language in DR Congo or in Lingala and Kikongo, the languages spoken in Kinshasa and Bas–Congo, respectively. Interviews typically lasted between 20 and 30 min. Multiple choice closed-ended questions were followed by open-ended questions to collect narrative responses. All answers were recorded in French by the interviewers.

### Ethics

The MATIAS study protocol was reviewed and approved by the ethics committee of the Kinshasa School of Public Health (University of Kinshasa) and by the ethics commission of both cantons of Basel, EKBB (*Ethikkommission beider Basel)* in Switzerland. Informed verbal consent was obtained from health care providers, patients and caretakers who participated in the study.

### Data processing and analysis

Quantitative data were entered electronically using Epi data 3.1 (Epidata Association; Odense, Denmark). After standard quality control checks, data were transferred to Stata version 12 (Stata Corporation; College Station, Texas) for analysis. Categorical variables were compared using pearson’s Chi square test or fisher’s exact test in case the expected value of any of the cells of the table was less than five. A *p* value ≤0.05 was considered statistically significant. Qualitative data were summarized in emerging themes which were coded and entered using Epi data 3.1. They are presented as proportions of different variables. Some answers are reported as narratives.

## Results

### Health care providers

Key results of interviews with health care providers are summarized in Table 1. A total of 31 health care providers were interviewed, whereby medical doctors and nurses accounted for 22.6 % (7/31) and 77.4 % (24/31) of the interviewed personnel, respectively. The median number of providers interviewed per health facility was four, ranging from three to five per facility. The majority of the personnel interviewed (28/31, 90.3 %) had more than 3 years of working experience, whilst three individuals (9.7 %) had 1–3 years experience. None of the health care providers interviewed had used injectable artesunate before the beginning of the study.

**Table 1:** Summary of interviews with health care providers

Question/parameter	Frequency	Percentage [95 % CI]
Did you spend more or less time to prepare artesunate compared to quinine (N = 24)?		
More time	3	12.5 [2.7, 32.4]
Same time	3	12.5 [2.7, 32.4]
Less time	18	75 [53.3, 90.2]
Did you find more or less difficult to prepare artesunate compared to quinine (N = 24)?		
More difficult	3	12.5 [2.7, 32.4]
Same difficulty	3	12.5 [2.7, 32.4]
Less difficult	18	75 [53.3, 90.2]
Most cited reasons to support ease of use of injectable artesunate (N = 24)		
Rapid way of administration	15	62.5 [40.6, 81.2]
No accidents related to infusion	11	45.8 [25.6, 67.2]
Reduced patient’s monitoring time	5	20.8 [7.1, 42.2]
Time to observe effects of injectable artesunate compared to quinine (N = 31)?		
Less time	29	93.6 [78.6, 99.2]
Same time	1	3.2 [0.1, 16.7]
More time	1	3.2 [0.1, 16.7]
Have you noticed any adverse events that you think could be related to artesunate (N = 31)?		
Yes	7	22.6 [9.6, 41.1]
No	24	77.4 [58.9, 90.4]
Which adverse events did you notice (N = 7)		
Decrease of haemoglobin level	5	71.4 [29.0, 96.3]
Shivering after drug injection	3	42.9 [9.9, 81.6]
Loss of weight	1	14.3 [0.4, 57.9]
If you noticed adverse events, would you say that: (N = 7)		
They are less than those observed with quinine	7	100
They are the same than those observed with quinine	0	0
They are more than those observed with quinine	0	0
Don’t know	0	0
Do you think that the workload has reduced with artesunate compare to quinine (N = 31)?		
The workload has diminished	26	83.9 [66.3, 95.4]
The workload is the same	4	12.9 [3.6, 29.8]
The workload has increased	1	3.2 [0.1, 16.7]
What is your level of satisfaction with injectable artesunate (N = 31)?		
Very satisfied	19	61.3 [42.2, 78.2]
Satisfied	12	38.7 [21.8, 57.8]
Dissatisfied	0	0
Most important reasons for being very satisfied/satisfied with injectable artesunate N = 31)		
Lack of adverse events	17	54.8 [36.0, 72.7]
Rapid action of the drug	15	48.3 [30.2, 66.9]
Easy way to prepare and administer	9	29.0 [12.4, 48.0]
Injectable artesunate more effective	9	29.0 [12.4, 48.0]
Work load reduction	8	25.8 [11.9, 44.6]

### Ease of use

Questions related to the handling of the drug were only asked to the 24 nurses who were responsible for the drug preparation and administration. Compared to quinine, eighteen (75 %) of all interviewed nurses reported to have spent less time to prepare and administer injectable artesunate, 3 (12.5 %) spent more time and 3 (12.5 %) said to have spent the same amount of time (Table 1). Eighteen (75 %) found it less difficult to prepare injectable artesunate compared to quinine, 3 (12.5 %) found it more difficult and three reported to have experienced the same level of difficulty (Table 1). All those who found it more difficult to prepare injectable artesunate compared to quinine specified that too many steps were needed in artesunate preparation. For patients above 50 kg body weight, a minimum of three vials are needed for a single dose and obviously this increased the time spent in drug preparation since each vial must be opened and reconstituted separately.

All interviewed nurses involved in the administration of the treatment found it less difficult to administer artesunate compared to quinine. The most important reasons cited by the respondents were the rapid means of administration (62.5 %), no accidents related to infusion (45.8 %) and the reduced patient monitoring time (20.8 %) (Table 1). None of the nurses interviewed encountered problems during drug preparation and drug administration.

### Perceived effectiveness and safety

Regarding the time to observe clinical effects, twenty nine (93.6 %) health workers reported that it took less time compared to quinine, 1 (3.2 %) estimated it took the same time and one (3.2 %) estimated it took more time (Table 1). 30 (96.7 %) health care providers reported to be very satisfied with the capacity of injectable artesunate to cure the symptoms of their patients compared to quinine, 1 (3.2 %) experienced the same satisfaction level, and none found injectable artesunate less satisfactory.

Seven (22.6 %) health workers reported to have noticed adverse drug reactions: The most common ones mentioned were a decrease of haemoglobin rate (71.4 %), shivering following the drug injection (42.9 %) and loss of weight (14.3 %). However, all the seven health care providers who reported adverse drug reactions answered that they were less frequent than those observed with quinine (Table 1).

### Patient management

The majority (96.8 %) of health care providers reported to have dedicated less time for patient monitoring after administration of artesunate compared to quinine. This proportion was not significantly different according to the type of health facility (hospitals versus health centres; p = 0.388 Fisher’s exact test). Of all health care providers interviewed, 26 (83.9 %) reported that the personnel workload had diminished with the use of injectable artesunate, 4 (12.9 %) reported the workload to be the same, while 1 (3.2 %) reported that the workload had increased (Table 1). The most important reasons for reported workload reductions were reduced patient monitoring time (88.5 %), saving of time by health personnel (80.7 %) and shorter treatment duration (15.4 %). A reason reported by one health care provider from Ngeba Health Centre for workload increase was increased patient monitoring time.

### Care providers general satisfaction

When health care providers were asked about their level of satisfaction with injectable artesunate they were either satisfied (38.7 %, 12/31) or very satisfied (61.3 %, 19/31) with the new treatment, with nobody giving negative feedback (Table 1). Reasons for being satisfied/very satisfied were lack of adverse events (54.8 %), rapid action of the drug (48.4 %), the easy way the drug is prepared and administered (29 %), injectable artesunate being more effective (29 %) compared to quinine and workload reduction (25.8 %). The level of satisfaction towards injectable artesunate was not significantly different among type of health facility (hospitals versus health centres; p = 0.452 Fisher’s exact test) and health care providers (medical doctors versus nurses; p = 0.384 Fisher’s exact test).

A nurse said about injectable artesunate: *“…I am very satisfied, it makes work easier, we have good time management, patient monitoring has been improved, there are no side effects, it has reduced mortality rate among children treated, the drug has attracted many patients to come to our health facility”.*

A medical doctor stated: *“very satisfied… it responds well, no side effects, but there’s a risk of a high cost because it is so precise and easier to use that such a product can only be more expensive than quinine… Good outcome after treatment.*”

### Patients or caretakers

Results of interviews with patients/caretakers are summarized in Table 2. A total of 134 patients/caretakers were interviewed (124 caretakers and 10 patients aged 12 years or older). There were more female (73.3 %, 96/134) than male (26.7 %, 35/134) respondents (p value <0.05). Of the 124 caretakers interviewed, 76 (61.3 %) were mothers of patients, 33 (26.6 %) were fathers, 14 (11.3 %) were other members of the family and the remaining 1 (0.8 %) was another member of the neighbourhood who accompanied a two-year old female patient at Ceco Health Centre.

**Table 2:** Summary of interviews with patients/caretakers

Question/parameter	Frequency	Percentage [95 % CI]
Time to observe effects of injectable artesunate compared to quinine (N = 134)?		
Less time	125	93.3 [87.6, 96.9]
Same time	8	6 [2.6, 11.4]
More time	1	0.7 [0.0, 4.1]
Have you noticed any adverse event that you think could be related to artesunate (N = 134)?		
Yes	46	34.3 [26.4, 43.0]
No	88	65.7 [57.0, 73.7]
If you noticed adverse events, would you say that: (N = 46)		
They are less than those observed with quinine	32	69.6 [54.3, 82.3]
They are the same than those observed with quinine	7	15.2 [6.3, 28.9]
They are more than those observed with quinine	1	2.1 [0.0, 11.5]
Don’t know	6	13.1 [4.9, 26.3]
If you had to make the choice in the future between quinine and artesunate, which one would you choose (N = 121)?		
Quinine	4	3.3 [0.9, 8.2]
Artesunate	117	96.7 [91.8, 99.1]
What is your level of satisfaction towards injectable artesunate (N = 134)?		
Dissatisfied	6	4.5 [1.7, 9.5]
Satisfied	66	49.2 [40.5, 58.0]
Very satisfied	62	46.3 [37.6, 55.1]
Most important reasons for choosing injectable artesunate instead of injectable quinine (N = 117)		
Rapid action	55	47.0 [37.7, 56.5]
No side effects	38	32.5 [24.1, 41.8]
Short treatment course	24	20.5 [13.6, 28.9]
Less side effects	14	12.0 [6.7, 19.3]
Rapid way of administration	13	11.1 [6.1, 18.3]
Short hospital stay	7	6.0 [2.4, 11.9]
More effective	5	4.2 [1.4, 9.7]

### Effectiveness and safety

With regards to the time needed for injectable artesunate to cure the symptoms of the patients, the large majority of respondents (93.3 %, N = 125) felt that it took less time compared to quinine, while 8 (6 %) respondents said it took the same time and 1 (0.7 %) more time. 46 (34.6 %) respondents reported to have noticed adverse events; asthenia (63 %) and loss of appetite (15.2 %) were the most common ones, while 87 (65.4 %) did not report any complication. The proportion of patients/caretakers reporting adverse events was not significantly different from that of care providers (X2 = 1.593, p value = 0.207). Statistical analysis showed no significant difference in the occurrence of adverse events between patients less than and more than 5 years of age (X2 = 0.162, p value = 0.687). Of those who reported to have noticed adverse events, 32 (69.6 %) considered that they were less than those observed with quinine, while 7 (15.2 %) and 1 (2.1 %) said respectively they are the same and more than those observed with quinine. 6 (13.1 %) did not know. The point made above on recall bias calls for some caution in the interpretation of these results.

### Satisfaction

Regarding general satisfaction towards the ability of injectable artesunate to cure the symptoms that motivated the patients’ consultation, the vast majority of patients/caretakers (95.5 %, N = 128) reported to have been either satisfied or very satisfied (Table 2). Six (4.5 %) reported being less satisfied than with quinine, of whom three reported persistent fever as a main reason for their dissatisfaction, while 2 (33.3 %) reported asthenia and dizziness. One respondent said he did not know what could be the long-term side effects of this new drug. Patients/caretakers level of satisfaction was not significantly different among type of health facility they consulted (p value = 0.46, Fisher’s exact test) and patient’s age (p value = 0.77 Fisher’s exact test). When asked if they would choose or recommend injectable artesunate over quinine again next time for themselves or a family member, the majority of respondents (97.7 %) said they would choose injectable artesunate. The most important reasons for choosing artesunate were rapid action (47 %), no or less adverse events (44.5 %), shorter treatment course and a shorter hospital stay (26.5 %) (Table 2).

A mother said: “*This is a short duration treatment, the symptoms disappear quickly. There is less manipulation compared to quinine, where you have to be in bed for 4 hours of infusion but with this treatment, just a few minutes of injection. This drug takes less time compared to quinine and there are no side effects. I think it is better suited to malaria treatment for children*”.

A young mother said: “*Very satisfied* - *After the first injection, my child was doing fine already. The fever had dropped quickly. The treatment duration is very short. We stayed for a short time at the hospital*”.

## Discussion

This study was designed to assess the feasibility and acceptability of the implementation of IV/IM artesunate from the perspective of care providers, as well as its acceptability (versus quinine treatment) from the perspective of the patients/caretakers. Results clearly show that use of injectable artesunate for the treatment of severe malaria in the context of the DRC is both feasible and well accepted. Patients/caretakers were very receptive to the new drug as they perceived it as being highly effective. Despite a few number of health providers reporting that several steps were needed in the preparation of artesunate, the handling of the drug was perceived to be easy. The vast majority of providers reported to have spent less time in this task. This is consistent with the results of quantitative measures of time and motion reported by Ferrari et al., which showed that the overall cumulative staff time dedicated to drug pre-administration tasks was 20 min for quinine compared to 13 min for artesunate. This difference is expected to improve in favour of the latter with health personnel gaining more experience [16].

Drug formulation had a significant impact on the duration of the preparation and administration. The drug used in the study was packaged in vials of 60 mg which, when reconstituted, was equivalent to 6 ml of solution for the intravenous route. For an average 60 kg body weight adult, this equates to prepare three vials and repeating three times all steps of preparation, resulting in a longer preparation time.

On the other hand, this drug formulation may cause significant drug wastage especially in small children who need small quantities. As the reconstituted solution is only stable for 1 hour, and since an opened vial cannot be reused, it is possible to lose up to more than half of the vial. In the context of limited resources, it is important that drug manufacturers develop adapted and easy-to-use forms of injectable artesunate.

Contrary to artesunate, the administration of quinine requires special precautions because of its potential toxicity, and close monitoring of the patient as the risk of incorrect dosage and severe side effects is high [9, 19–21]. This leads to a reduced patient monitoring time with the use of injectable artesunate which may explain the reported reduction of personnel workload which in turn has the potential to improve the quality of care.

The superior efficacy of injectable artesunate compared to intravenous quinine in the management of severe malaria has been demonstrated in clinical trials [6–8]. Because of its small-scale nature based on purposive sampling, this study cannot draw a conclusion on the effectiveness of injectable artesunate. However, both health care providers and patients/caretakers perceive artesunate to be highly effective.

The findings from this study are consistent with what is known so far about the better short-term safety of artesunate compared to quinine [6] [22]. Patients/caretakers did not report significant adverse event, the commonly reported adverse events (asthenia and loss of appetite) may be disease induced.

The most common adverse events reported by health workers was a decrease in haemoglobin, a fact supporting recent findings on the occurrence of delayed anaemia after parenteral artesunate for severe malaria [13–15]. However, the training received by health workers before the implementation of artesunate had an emphasis on the monitoring of adverse events and especially a drop in haemoglobin, and this may have influenced the frequency of reporting. The results of this study cannot be used to draw conclusion on the safety of intravenous artesunate, but rather only as supportive evidence to the acceptability of the new treatment.

The design without concurrent controls, the relatively small scale of the study and the purposive sampling constitute a limitation to the generalizability of the findings. The majority of interview questions were comparative between quinine and artesunate and the time between interviews and prior experience with quinine treatment was not recorded, this might have led to a recall bias, especially for interviews with patients/caretakers. Courtesy bias in respondents’ answers might be possible as the drug cost was free for patients and interviewed health care providers were involved in the MATIAS project. In order to minimize this, interviews were conducted by independent interviewers recruited from the local community.

One of the major challenges in switching from quinine to injectable artesunate may be the reluctance of health care workers to switch to a new treatment [23]. In this study, the majority of health care providers were not aware of the latest evidence on safety and efficacy, and they are very familiar with quinine treatment. Hence, it is important to promote the benefits of injectable artesunate among health workers and train them well in the use of the new treatment. The new treatment guidelines should be included as soon as possible in the training curricula in medical and nursing schools, and public awareness of the new drug should be raised through effective communication channels.

## Conclusions

The findings from this study showed that the use of injectable artesunate for the management of severe malaria in hospitals and health centres of the DRC is feasible and acceptable to both care providers and patients/caretakers. The handling of the drug was perceived to be easy. Injectable artesunate was perceived to be very effective and safe. Training of health personnel is a key factor for a successful implementation. This study provides for the first time operational evidence to support the roll out of injectable artesunate in the DRC.
